# CT Imaging Biomarkers Predict Clinical Outcomes After Pancreatic Cancer Surgery

**DOI:** 10.1097/MD.0000000000002664

**Published:** 2016-02-08

**Authors:** Liang Zhu, Xiaohua Shi, Huadan Xue, Huanwen Wu, Ge Chen, Hao Sun, Yonglan He, Zhengyu Jin, Zhiyong Liang, Zhuoli Zhang

**Affiliations:** From the Department of Radiology (LZ, HX, HS, YH, ZJ); Department of Pathology (XS, HW, ZL); Department of Surgery, Peking Union Medical College Hospital (GC), Beijing, China; Department of Radiology, Northwestern University, Chicago, IL (ZZ); and Tianjin Key Laboratory of Cardiovascular Remodeling and Target Organ Injury, Pingjin Hospital Heart Center, Tianjin, China (ZZ).

## Abstract

This study aimed to determine whether changes in contrast-enhanced computed tomography (CT) parameters could predict postsurgery overall and progression-free survival (PFS) in pancreatic cancer patients. Seventy-nine patients with a final pathological diagnosis of pancreatic adenocarcinoma were included in this study from June 2008 to August 2012. Dynamic contrast-enhanced (DCE) CT of tumors was obtained before curative-intent surgery. Absolute enhancement change (AEC) and relative enhancement change (REC) were evaluated on DCE-CT. PFS and overall survival (OS) were compared based on CT enhancement patterns. The markers of fibrogenic alpha-smooth muscle antigen (α-SMA) and periostin in tumor specimens were evaluated by immunohistochemical staining. The χ^2^ test was performed to determine whether CT enhancement patterns were associated with α-SMA-periostin expression levels (recorded as positive or negative). Lower REC (<0.9) was associated with shorter PFS (HR 0.51, 95% CI: 0.31–0.89) and OS (HR 0.44, 95% CI: 0.25–0.78). The α-SMA and periostin expression level were negatively correlated with REC (both *P* = 0). Among several CT enhancement parameters, REC was the best predictor of patient postsurgery survival. Low REC was associated with a short progression-free time and poor survival. The pathological studies suggested that REC might be a reflection of cancer fibrogenic potential.

## INTRODUCTION

Pancreatic adenocarcinoma (PDA) is the fourth leading cause of cancer related mortality in United States.^1^ it is considered a highly lethal disease, since infiltration to peripancreatic structures and invasion to major vessels occurs relatively early, often “silently,” and only less than 20% patients were operable at the time of diagnosis.^2^ The overall 5-year survival for all pancreatic cancer patients is less than 5%.^3^ For patients who have their tumor resected, the 5-year survival could roughly achieve 20%.^4–6^ However, most patients who receive curative-intent surgery still failed to achieve long-term survival, since recurrence or distant metastasis occurs soon after the surgery.^7^ Therefore for operable patients, further risk stratification is necessary. It is important to identify presurgical prognostic factors, which could predict the postsurgery progression-free time interval, as well as total life expectancy. Such information may benefit an individualized and more efficient treatment strategy.

Contrast-enhanced CT (CECT) using a defined pancreas protocol has become widely accepted for pancreatic cancer staging and resectability assessment.^8^ On dynamic enhanced CT, these tumors usually enhance poorly, compared to the uninvolved parenchyma.^9,10^ Several studies have investigated the relationships between CT enhancement patterns and the clinical features of pancreatic cancer patients.^11–15^ However, the classifications of “enhancement patterns” were subjective and an identical criteria was lacking. Moreover, most previous studies have not directly correlated CT enhancement patterns with patient prognosis. Recently, Fukukura et al^16^ reported that, in 92 pancreatic cancer patients, higher contrast enhancement in the pancreatic phase was significantly associated with longer overall survival (OS). This study included both operable patients and patients with more advanced disease, who received treatments other than surgery, and the progression-free survival (PFS) time after treatment was not evaluated. Currently, there has been only limited evidence regarding whether CT enhancement pattern could predict the outcomes of pancreatic cancer patients, particularly those receiving curative-intent surgery, while for the latter group, prognostic information is of great clinical importance, and differ greatly from those who have metastases at diagnosis and receive treatments other than surgery.^17^

It is well known that pancreatic cancer often elicits desmoplastic reactions, causing dense fibrotic deposition in the intratumoral and juxtatumoral stroma, which contributes to its unique CT enhancement pattern.^9,13^ More recently, the role of activated stellate cells in pancreatic cancer development has been revealed.^18,19^ The significant fibrogenic potential originates from activated stellate cells in the pancreatic cancer parenchyma and their profound interaction with cancer cells, facilitating proliferation. Α-SMA and periostin are biomarkers for activated pancreatic stellate cells, and they have found to be overexpressed significantly in pancreatic cancer tissues.^20–22^ Whether the extent of satellite cell activation is associated with CT enhancement pattern remains unknown.

Therefore, the aims of our study were to evaluate different parameters reflecting CT enhancement patterns in patients who receive curative-intent surgeries for pancreatic cancer, to analyze their associations with postsurgery outcomes, and to investigate correlation between CT enhancement patterns and tumor fibrogenic potentials.

## METHODS

### Ethics Statement

This retrospective study was approved by the institutional review board of Peking Union Medical College Hospital. Written informed consent was obtained before collection. The histological types were assigned according to the criteria of the WHO classification system. The institutional review board approval can be found in Protocols S1.

### Patient Population

This retrospective study was approved by the institutional review board of Peking Union Medical College Hospital. From June 2008 to August 2012, 679 consecutive patients underwent contrast-enhanced pancreatic CT at our institution for suspected pancreaticobiliary malignancies. Among this population, the study population was selected using the following inclusion criteria: no evidence of metastases found by the preoperative evaluation; curative-intent surgery performed; pathologic diagnosis of PDA established after surgery. Patients with a history of neo-adjuvant chemotherapy or radiation therapy before CT scanning were excluded (n = 24).

The final study population consisted of 79 patients (43 men and 36 women, mean age, 59.0 years old, range, 33–80 years old). All patients received curative-intent surgery within 30 days (2–27 days, average 6.7 days) after CT scan.

The clinical information and pathological results of these patients were collected from a prospective database. Patient gender, age, surgical margin, tumor location, size, American Joint Committee on Cancer (AJCC) tumor staging and pathological differentiation were recorded and analyzed in all of the patients. Surgical margin was determined by pathological examination of then bloc surgical specimen following AJCC classifications. R0 denotes negative surgical margin, R1 denotes microscopic margin involvement and R2 denotes macroscopic margin involvement. R1 and R2 status were classified as positive margins. All the patients were followed up after surgery. PFS and OS times were recorded.

### Image Acquisition

CT examinations were performed on 64- or 128-slices CT scanners (Somatom Sensation 64 or Somatom Definition Flash, Siemens Medical Solutions, Forchheim, Germany). The scanning parameters were as follows: tube voltage, 120 kVp; effective amperage settings, 150 mAs; gantry rotation time, 0.5 seconds; table increment, 46.8 mm per rotation; field of view: 50 cm × 50 cm; and matrix 512 × 512. Images were reconstructed with a section thickness of 5.0 mm and reconstruction intervals 0 mm.

A total of 1.5 mL of nonionic contrast material (iopromide, 370 mg of iodine per milliliter, Ultravist 370, Schering, Berlin, Germany) per kilogram of body weight was injected at a rate of 3.0 mL/second, using an automatic power injector. Bolus-tracking was used, and the pancreatic parenchymal (PP) phase scan was initiated 5 seconds after enhancement of the descending aorta reaching 100 HU. The portal venous (PV) phase scan was initiated 25 seconds after the PP phase acquisition.

### CT Enhancement Pattern Evaluation

The CT images were reviewed on Picture Archiving and Communicating System (PACS) and tumor resectability was retrospectively analyzed by 2 radiologists (with 12 and 9 years of experience in pancreatic imaging, respectively). Both radiologists were aware of the diagnosis of pancreatic cancer and the tumor location, while they were blinded to other pathological information and patient's outcomes. The tumor resectability was categorized according to the National Comprehensive Cancer Network (NCCN) 2009 criteria as: resectable; borderline resectable; and unresectable. Any disagreement was discussed until consensus was reached.

**FIGURE 1:**
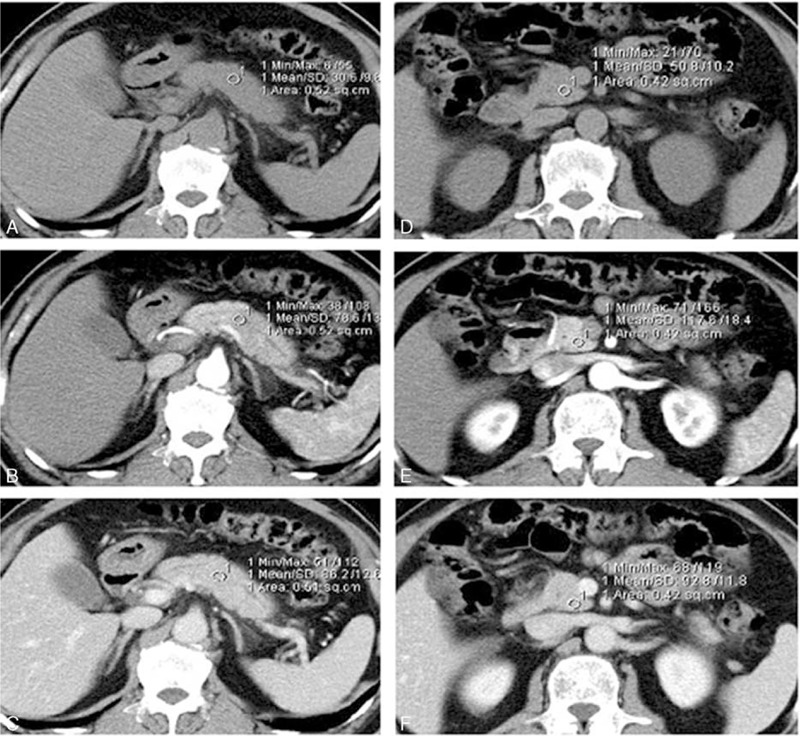
A 53-year-old woman with an isoattenuating mass in the pancreatic body. Pathology confirmed pancreatic ductal adenocarcinoma. A circular ROI was placed within the tumor on noncontrast images (A). The ROI was copied and pasted to the same position on PP (B) and PV (C) phase images, which were manually coregistered. ROIs of similar sizes were placed within normal parenchyma on noncontrast (D), PP (E), and PV (F) phase images.

**FIGURE 2:**
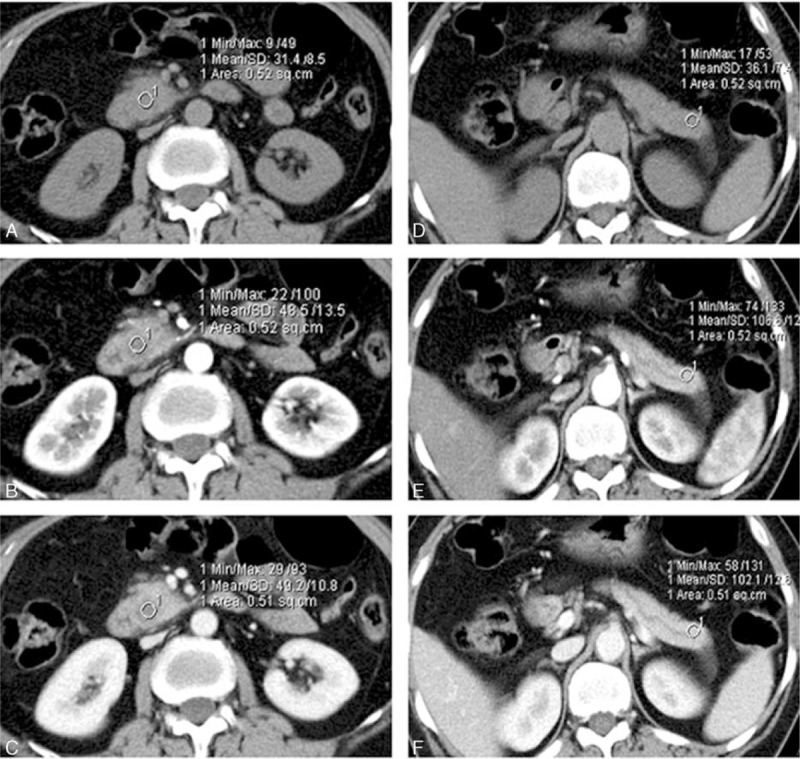
A 47-year-old man with pancreatic adenocarcinoma confined in the pancreatic uncinate. A ROI was placed within the tumor on carefully coregistered noncontrast images (A) and PP (B) and PV (C) phase images; another circular ROI of similar size was placed within the normal parenchyma on noncontrast (D), PP (E), and PV (F) phase images.

CT images were then retrieved to a postprocessing workstation (Syngo Dual Energy MMWP Workstation, Siemens) and the CT attenuation values of both cancer tissue and normal parenchyma were measured on both PP phase and PV phase images by one radiologist (with 12 years of experience). A circular region-of-interest (ROI) ranging from 30 to 50 mm^2^ was placed within the tumor. Care was taken to avoid proximity to blood vessels and necrotic tissue. Another circular ROI of identical size was placed in the parenchyma at least 1.0 cm away from the margin of the tumor. Care was taken to avoid proximity to blood vessels and dilated ductal structures. For isoattenuating tumors, the tumor location was identified with reference to pathological reports. Whenever possible, ROI measurements of both upstream and downstream pancreatic parenchyma were obtained. Images of the PP phase and PV phase were carefully coregistered by identifying the relative positions of abdominal organs with minimal displacement due to breathing movement (Figures 1 and 2).

Tumor contrast enhancement in the PP phase and PV phase was calculated by subtraction of the tumor attenuation value on nonenhanced images. Two additional parameters of CT enhancement patterns were calculated: absolute enhancement change (AEC) and relative enhancement change (REC). AEC was defined as the tumor attenuation change during the PP phase and PV phase, and REC was defined as the proportion of attenuation change between the tumor and pancreatic parenchyma during the PP phase and PV phase. The 2 values were calculated as follows: 
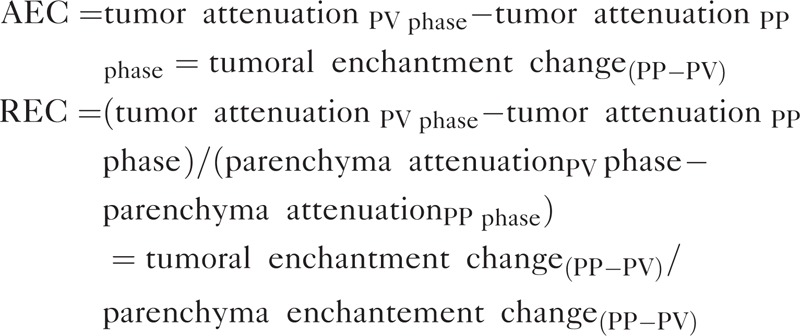


### Immunohistochemical Staining

Immunohistochemical (IHC) staining was performed using the EnVision system (DAKO, Glostrup, Denmark). Serial 5-μm-thick sections were cut from formalin-fixed and paraffin-embedded tumor blocks and were dewaxed in xylene, rehydrated through sequential changes of alcohol, and then antigen retrieved in 0.01 M citrate buffer with pH 6.0, at 90°C for 20 minutes. After washing with phosphate-buffered saline (PBS), the tissue sections were incubated with fresh 3% hydrogen peroxide for 20 minutes at room temperature. The sections were blocked with 20% goat serum for 30 minutes and incubated with α-SMA or periostin primary antibody (1:200 dilution; Abcam, Cambridge, UK) for 2 hours. The sections were then incubated with a polymer HRP secondary antibody (DAKO). Immunostaining was finally developed with 3,3′-diaminobenzidine (DAB). Positive and negative controls were run as appropriate.

### Pathological Analysis

**FIGURE 3:**
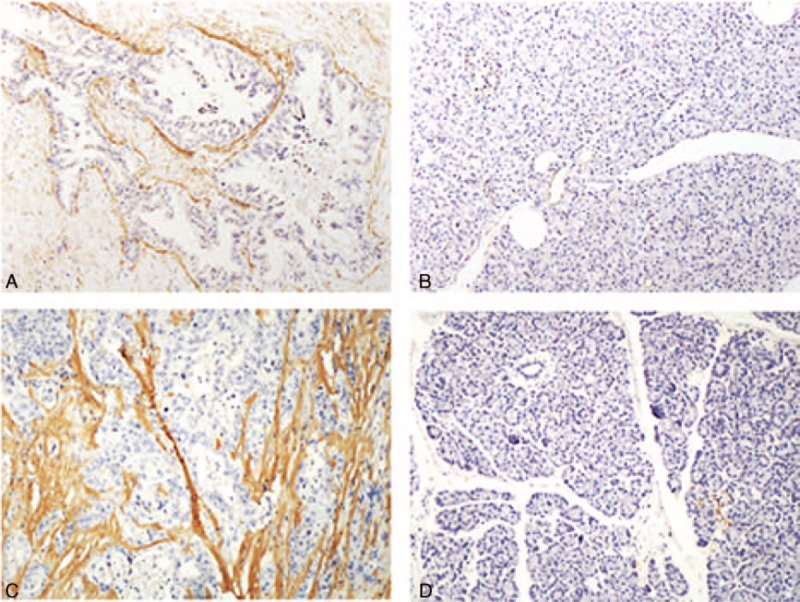
Representative examples of positive and negative staining of α-SMA (A and B, respectively) and periostin (C and D, respectively) in a resected pancreatic cancer tissue specimen.

The sections were assessed independently by 2 pathologists (both with 6 years of pathological experience) blinded to the clinical and imaging data. The scoring for each section was determined in consensus. The expected staining pattern for antiperiostin and α-SMA is cytoplasmic. The IHC staining results for antiperiostin and α-SMA were evaluated at the stromal tissue immediately adjacent to the neoplastic epithelial cells (within 100 μm) of infiltrating carcinoma, using a 4-tiered scale that was based on the percentage of immunoreactive tumor epithelial cells in the examined tissue irrespective of staining intensity: 0, 0% positive; 1, 1% to 25% positive; 2, 26% to 49% positive; or 3, ≥50% positive.^23^ Vascular smooth muscle cells served as an internal positive control for α-SMA immunostaining. Representative examples of positive and negative staining are shown in Figure 3.

### Statistical Analysis

For CT enhancement pattern analysis, the patients were arbitrarily divided into 2 groups based on a cut-off of the median value.^15^ The effects on survival of different variables, including patient age, sex, tumor enhancement pattern, tumor location, size, surgical margin, and pathological differentiation, were evaluated using the log-rank test. The adjusted hazard ratio (HR) was calculated with a Cox proportional hazards model using the enter method. The input variables for multivariable analysis were those found to be statistically significant on univariate analysis. Corresponding survival curves were estimated by the Kaplan–Meier method.

To assess whether expression of tumor fibrogenic parameters α-SMA and periostin were inter-correlated, Spearman rank correlation test was performed, as the intensity scores were ordinal.

The χ^2^ test was performed to determine whether CT enhancement patterns were associated with α-SMA and periostin expression levels (recorded as positive or negative).

All of the statistical analyses were conducted using SPSS software, version 19.0 (SPSS for Windows, SPSS, Inc., IBM, Armonk, NY). Results were considered to be significant at a 2-sided 5% significance level (*P* < 0.05).

## RESULTS

### Patient Characteristics and Follow-Ups

A total of 79 patients were analyzed. The clinicopathological characteristics are summarized in Table 1.

**TABLE 1:**
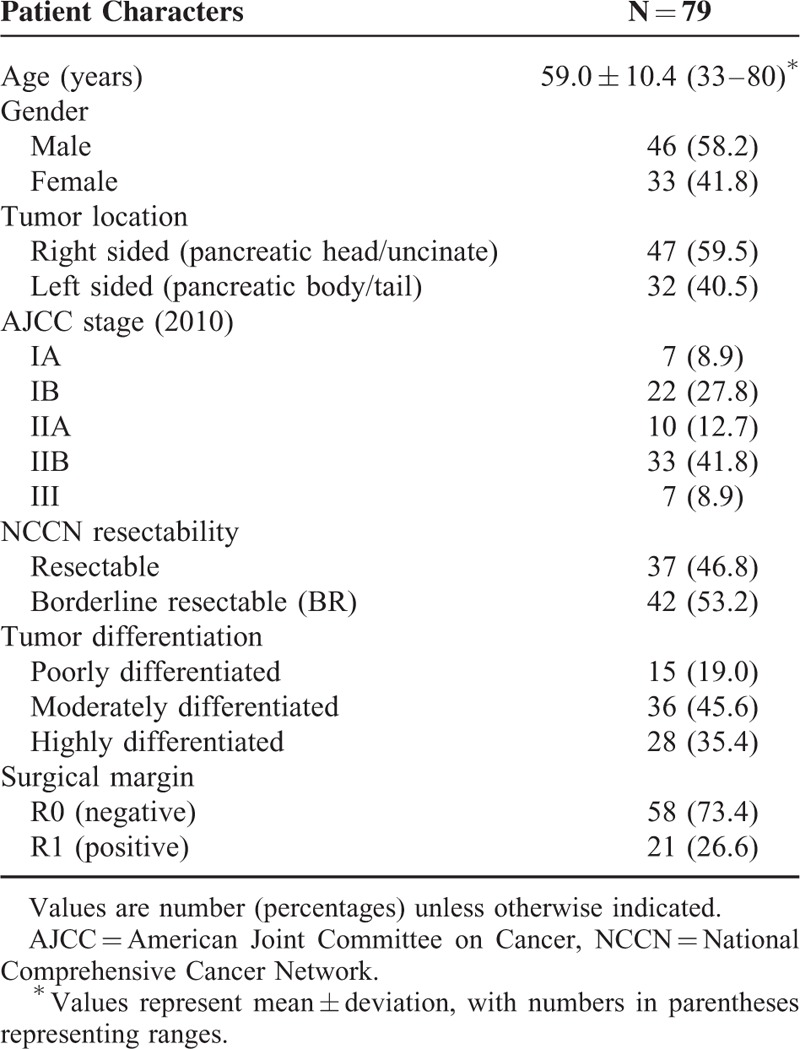
Patient Characteristics

The median follow-up of PFS was 9.0 months (range, 1–58 months). Disease progression was observed in 65 patients (82.3%). The median PFS time was 10.0 months (95% confidence interval: 8.1, 11.9 months).

The median follow-up of OS was 18.0 months (range, 3–53 months). Outcome events were observed in 52 patients (65.8%). The median OS time was 20.0 months (95% confidence interval: 17.5, 22.5 months).

### CT Enhancement Patterns

The CT enhancement patterns of all patients are summarized in Table 2. For the majority of patients, both the tumor (86.1%) and normal pancreatic parenchyma (87.3%) followed a pattern of increasing enhancement from the PP phase to the PV phase.

**TABLE 2:**
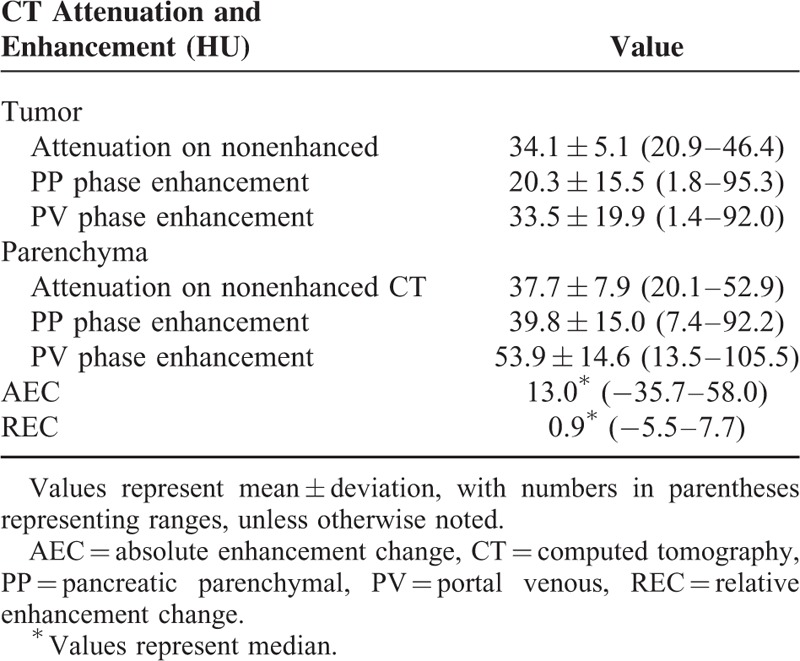
CT Enhancement Pattern of the Pancreatic Cancer and Parenchyma

The median AEC was 13.0 (range: −35.7 to 58.0; interquartile range: 6.8 to 23.4). The median was used as a cut-off, dividing patients into low-AEC (<13.0, n = 39) and high-AEC groups (≥13.0, n = 40).

The median REC in all of the patients was 0.9 (range: −5.5 to 7.7; interquartile range: 0.41 to 0.90). Using the median value as a cut-off, the patients were divided into low-REC (<0.9, n = 39) and high-REC (≥0.9, n = 40) groups.

### Survival Analysis

Univariate analysis of variables including clinicopathological factors and enhancement pattern are shown in Table 3 (PFS) and Table 4 (OS). Among clinicopathological factors, patient age (*P* = 0.22), sex (*P* = 0.70), tumor location (*P* = 0.15), size (*P* = 0.38), and resectability (*P* = 0.31) were not significantly associated with PFS, whereas tumor differentiation (*P* < 0.01) and surgical margin (*P* = 0.01) had significant influence on PFS. Among enhancement parameters, tumor enhancement in the PP phase and in the PV phase and AEC (tumor enhancement change from the PP to PV phase) were not significantly associated with PFS (*P* = 0.12, 0.06, and *P* = 0.40, respectively). REC was the only enhancement parameter with significant influence on PFS (*P* = 0.01).

**TABLE 3:**
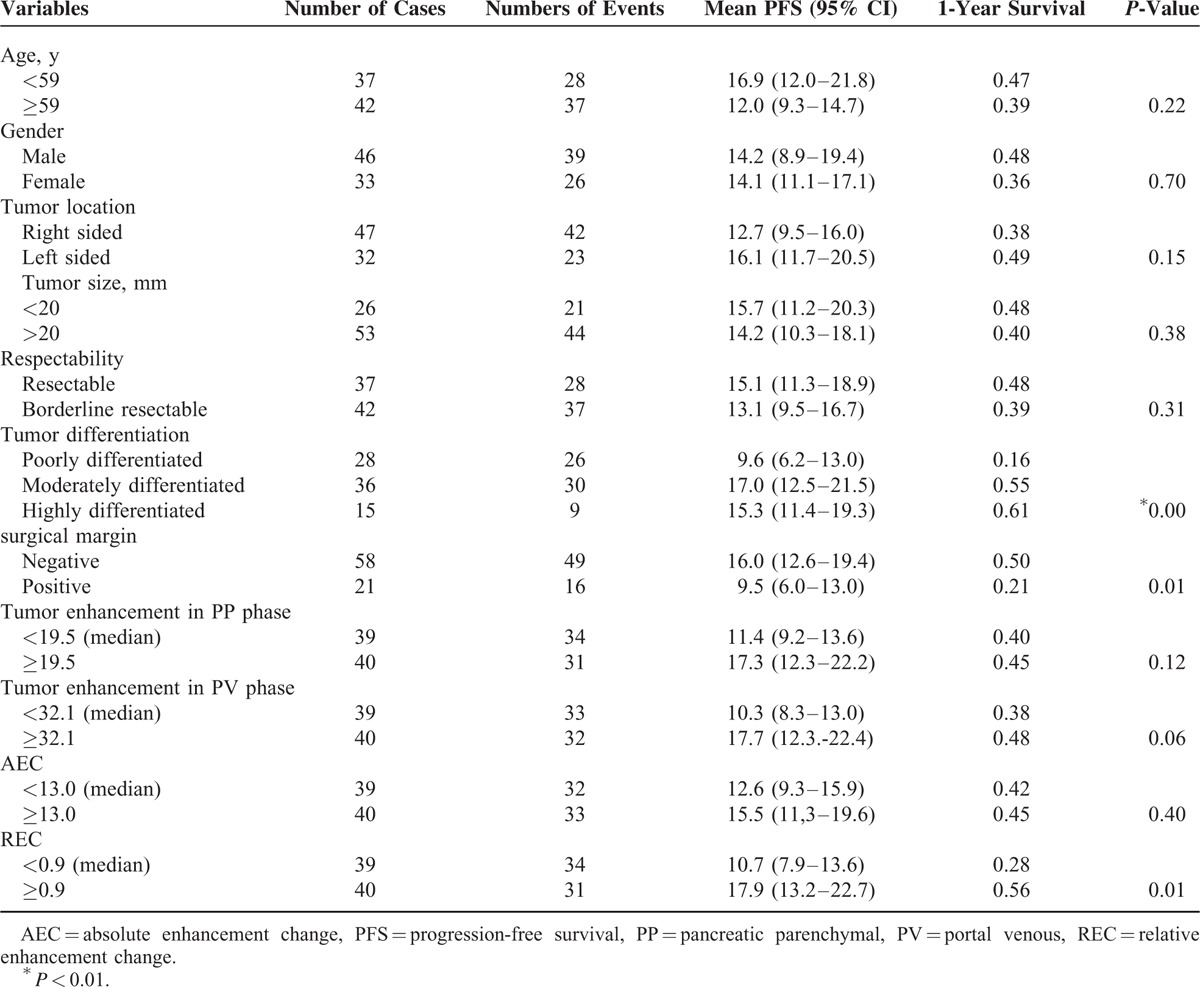
Univariate Analysis for PFS in 79 Resected Pancreatic Cancer Patients

**TABLE 4:**
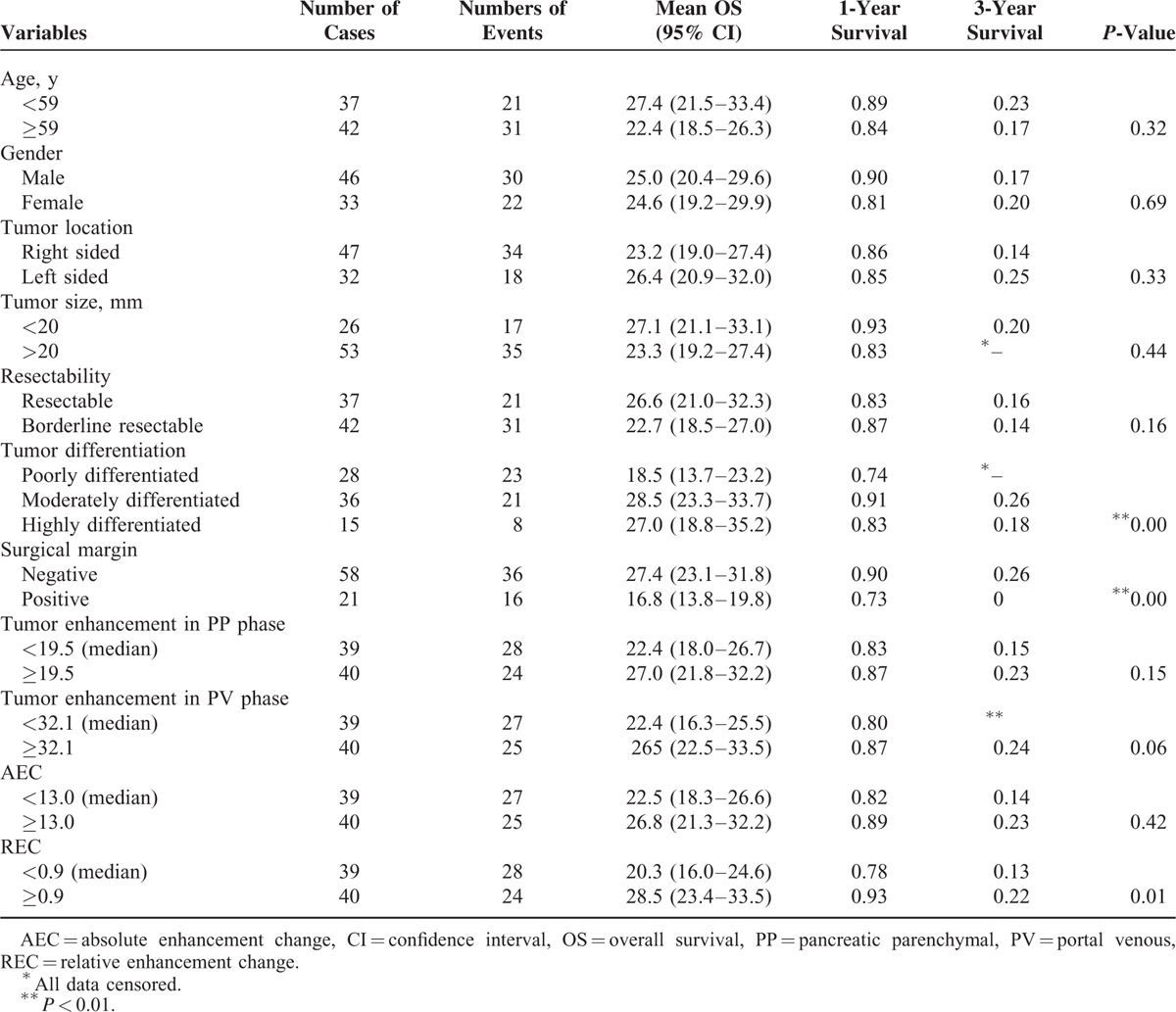
Univariate Analysis for OS in 79 Resected Pancreatic Cancer Patients

**FIGURE 4:**
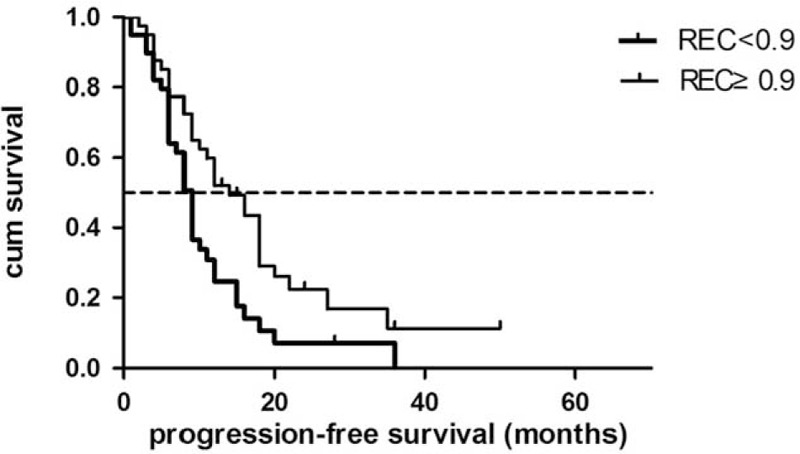
Progression-free survival for low-REC patients and high-REC patients.

In multivariate analysis, REC, resection margin, and tumor differentiation remained independent prognostic factors of PFS (Table 5). Low REC (<0.9) was consistent with a shorter PFS time (*P* = 0.01). Patients with low REC had a median PFS of 10.7 months (95% CI: 7.9–13.6 months), whereas patients with high REC had a median PFS of 17.9 months (95% CI: 13.2–22.7 months) (Figure 4).

**TABLE 5:**
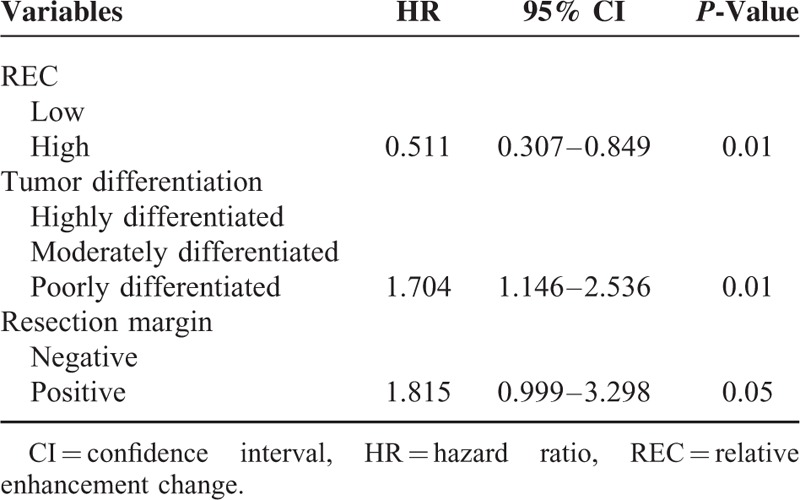
Multivariate Analysis for PFS in 79 Resected Pancreatic Cancer Patients

Among clinicopathological factors, patient age (*P* = 0.32), sex (*P* = 0.69), tumor location (*P* = 0.33), tumor size (*P* = 0.44), and tumor resectability (*P* = 0.16) were not significantly associated with OS, whereas tumor differentiation (*P* < 0.01) and surgical margin (*P* < 0.01) had significant influences on OS. Among enhancement parameters, tumor enhancement in the PP phase and in the PV phase and AEC were not significantly associated with OS (*P* = 0.15, 0.06, and *P* = 0.42, respectively). REC was the only enhancement parameter with a significant influence on OS (*P* = 0.01).

**FIGURE 5:**
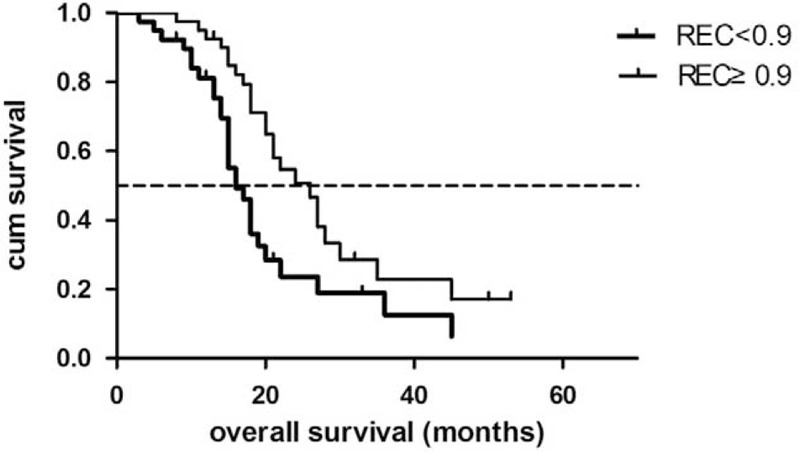
Overall survival for low-REC patients and high-REC patients.

REC, resection margin, and tumor differentiation were identified as independent prognostic factors for OS by multivariate Cox regression (Table 6). Patients with a low REC had a median OS of 20.3 months (95% CI: 16.0–24.6 months), whereas patients with high REC had a median OS of 28.5 months (95% CI: 23.4–33.5 months) (Figure 5).

**TABLE 6:**
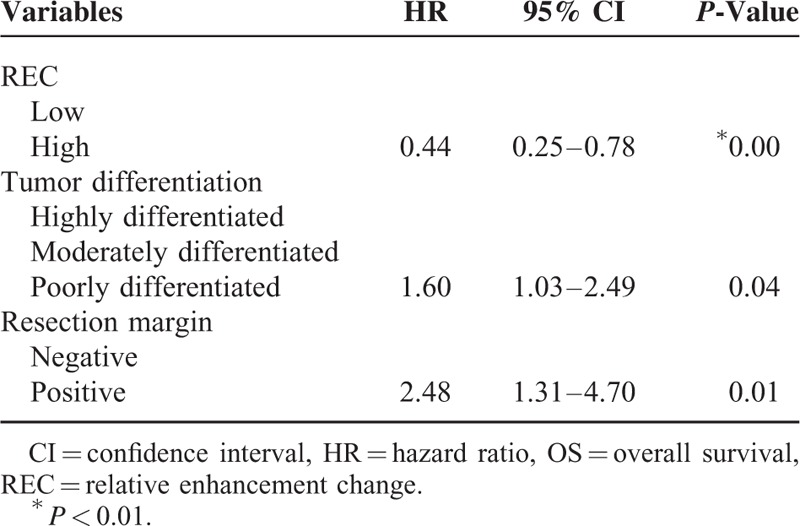
Multivariate Analysis for OS in 79 Resected Pancreatic Cancer Patients

### Relationship Between CT Enhancement Pattern and Tumor Fibrogenic Markers

The tumor fibrogenic markers α-SMA and periostin protein expression were significantly associated with one another (ρ = 0.549, *P* < 0.01). The χ^2^ test showed significant correlation between REC and α-SMA (*P* = 0.01), as well as between REC and periostin (*P* = 0.01).

## DISCUSSION

In our study, several quantitative parameters reflecting pancreatic cancer enhancement pattern were investigated from the perspective of postsurgical outcome prediction. We found that REC was the best imaging prognostic factor, whereas enhancement of the tumor itself in the PP or PV phase was not directly predictive for disease progression or OS.

Previous studies have provided direct and indirect evidence of potential correlations of CT enhancement patterns of pancreatic cancer with patient outcomes. The study undertaken by Yoon et al^9^ suggested that pancreatic cancers that were isoattenuating on initial CT imaging showed decreased attenuation on follow-up images 6 months later. In addition, patients with hypoattenuating tumors were more prone to experience liver metastasis. Thus, the low enhancement of the tumor might be a reflection of a later disease stage and tumor composition changes. Scialpi et al^24^ attempted to correlate the CT enhancement patterns of small PDA with histologic features. Their study of 38 patients with small PDA showed that quantitative analysis could better detect lesions, although this method was less sensitive for patients with severe fibrous stroma due to chronic pancreatitis. The enhancement patterns of PDA on contrast-enhanced ultrasonography also showed their correlations with histology grade and several prognostic factors.^14,25^

The typical progressively delayed enhancement pattern is unique for pancreatic cancer and distinguishes it from other solid pancreatic tumors, which reflects the profuse fibrotic component within and adjacent to the tumor. However, the parameters reflecting enhancement patterns are not consistent. Some authors have reported “enhancement patterns” in a relatively subjective manner.^26,27^ Recently, Fukukura et al^15^ reported that tumor enhancement in the PP phase was the best outcome predictor for patient with pancreatic ductal adenocarcinoma. In a subgroup analysis of 29 patients who received curative-intent surgery, poor tumor enhancement in the pancreatic parenchyma phase was also significantly correlated with poor postsurgical OS. These results were not reproduced in our study of 79 surgical cases. This discrepancy might be explained by the different characteristics of the included patients regarding tumor stage and location, as well as differences in CT scanning parameters, including timing and injection rate of contrast agents. However, as observed in many previous studies, including ours, the values of tumor enhancement in any phases showed a broad range among different patients,^13–15^ which could be explained by the individual variations in abdominal visceral perfusion after contrast media injection.^28^ Moreover, for pancreatic cancer, the uninvolved part of the pancreas, which appears “normal,” often has a background of chronic pancreatitis or anaplastic changes on pathological examination.^11,14,24^ In addition, the pancreas is a retroperitoneal organ, which is in close proximity to the celiac artery and superior mesenteric artery. Pancreatic cancer often has early infiltration or perivascular desmoplastic reactions, which can influence its direct blood supply. All of these factors could contribute to the enhancement patterns of pancreatic cancer, and they should be considered for analysis. REC uses the enhancement change of the uninvolved pancreatic parenchyma as the denominator. In this manner, the background enhancement difference caused by bolus timing and the pancreatic blood supply could be partially compensated, whereas the specific differences caused by special cancer tissue components or by the unique tumor microenvironment are intensified.

Although tumor size, tumor location, and resectability have been found to be independent prognostic determinants in previous studies,^6,29–35^ our study did not show statistical significance regarding the above variables. Small pancreatic cancer (≤2 cm) is concordant with a relatively early stage in disease development, and a better outcome would be expected. However, only operable patients were enrolled in this study, and most of them had relatively small tumors, with an average tumor size of 2.4 cm. Cancers originating from the pancreatic body and tail have been associated with worse prognoses because of a lack of early symptoms and local invasiveness being common at initial diagnosis. However, in our study, a considerable number of patients with pancreatic body/tail cancer were identified in relatively early stages, which could likely be explained by institution bias. The survival differences between the resectable and borderline resectable groups were not significant as expected. Tumors with direct abutment of the major vessels but no obvious impingement fell into the borderline resectable group according to the NCCN criteria^36^; however, only a few (n = 6) of the patients included in this study underwent vessel reconstruction. Moreover, postsurgical treatment was not included in our Cox regression model, which might have confounded the significance of intergroup differences in resectability.

Previous research into the potential correlations between CT enhancement patterns and pathological features of pancreatic cancer has moved in two main directions: microvessel density (MVD) and tumor fibrogenesis. Wang et al correlated the CT enhancement degree of pancreatic cancer with pathological grading and MVD, and they showed that the extent of enhancement in the pancreatic phase was inversely proportional to the degree of malignancy of pancreatic cancer and to the MVD number in cancer tissue.^37^ A study of 21 pancreatic cancer patients undertaken by Hata et al^14^ demonstrated correlations of CT enhancement patterns with tumor vascularity and fibrosis. Tumors with more fibrosis and higher vascularity were found to have a higher contrast effect through all of the arterial, portal venous, and delayed phases. In addition, they reported that tumors with liver metastasis tended to be less fibrotic than tumors without liver metastasis. The significant fibrogenic potential of pancreatic cancer arises from activated stellate cells in the parenchyma, which have profound interactions with cancer cells, facilitating proliferation. α-SMA and periostin are generated by activated pancreatic stellate cells,^18,20–22,38–40^ and they have served as fibrogenic markers, although direct evidence of their correlations with patient outcomes have not yet been established. Our study provides further supportive evidence for the correlation of CT enhancement pattern with tumor fibrogenic potential. Pancreatic cancer that enhanced poorly tended to have higher fibrotic content and a less favorable prognosis.

Our study had several limitations. First, our patient group was relatively small, and there were wide and overlapping confidence intervals around the median survival times, which should be considered in data interpretation. Second, our patients received various postsurgical treatments, which were difficult to record and categorize accurately, and we did not add these variables to our Cox multivariate model, which might have caused considerable confounding.

## CONCLUSIONS

REC is a quantitative parameter of the pancreatic cancer enhancement pattern. It can be evaluated on preoperative CT, and it was an independent prognostic factor for postsurgical outcomes, including PFS and OS. Patients with low REC (≤0.9) had a higher risk of short-term recurrence and a poorer opportunity to achieve long-term survival after surgery. REC was negatively correlated with α-SMA and periostin expression levels in surgical specimens, suggesting that REC might be a reflection of tumor fibrogenic potential.
