# CT Imaging Biomarkers Predict Clinical Outcomes After Pancreatic Cancer Surgery: Erratum

**DOI:** 10.1097/01.md.0000482791.58629.ce

**Published:** 2016-04-18

In the article “CT Imaging Biomarkers Predict Clinical Outcomes After Pancreatic Cancer Surgery”,^1^ which appeared in Volume 95, Issue 5 of *Medicine*, Dr. Zhang was incorrectly listed as a corresponding author originally. The article has since been corrected online.
